# Bet-hedging and variability in plant development: seed germination and beyond

**DOI:** 10.1098/rstb.2023.0048

**Published:** 2024-04-22

**Authors:** Katie Abley, Rituparna Goswami, James C. W. Locke

**Affiliations:** The Sainsbury Laboratory, University of Cambridge, Cambridge, Cambridgeshire CB2 1LR, UK

**Keywords:** bet-hedging, noise in gene expression, germination, computational models, single-cell timelapse microscopy

## Abstract

When future conditions are unpredictable, bet-hedging strategies can be advantageous. This can involve isogenic individuals producing different phenotypes, under the same environmental conditions. Ecological studies provide evidence that variability in seed germination time has been selected for as a bet-hedging strategy. We demonstrate how variability in germination time found in Arabidopsis could function as a bet-hedging strategy in the face of unpredictable lethal stresses. Despite a body of knowledge on how the degree of seed dormancy versus germination is controlled, relatively little is known about how differences between isogenic seeds in a batch are generated. We review proposed mechanisms for generating variability in germination time and the current limitations and new possibilities for testing the model predictions. We then look beyond germination to the role of variability in seedling and adult plant growth and review new technologies for quantification of noisy gene expression dynamics. We discuss evidence for phenotypic variability in plant traits beyond germination being under genetic control and propose that variability in stress response gene expression could function as a bet-hedging strategy. We discuss open questions about how noisy gene expression could lead to between-plant heterogeneity in gene expression and phenotypes.

This article is part of a discussion meeting issue ‘Causes and consequences of stochastic processes in development and disease’.

## 1. Introduction

Plant development occurs mostly post-embryonically and is well known for its sensitivity and plasticity to the environment [1]. In most plant species, many aspects of development, from the control of stem elongation, to root growth, shoot branching and leaf shape are modulated by the environment, such that isogenic (i.e. close to genetically identical) seeds sown in different environments will give rise to phenotypically distinct plants. This developmental plasticity can be adaptive, involving the plant sensing environmental cues and modulating its development accordingly, such that its resulting phenotype promotes reproductive success in the conditions it finds itself in [1,2]. Phenotypic plasticity works well when the future environment can be predicted from current environmental cues [3,4].

However, when the environment is unpredictable, current environmental cues are not reliable predictors of the future environment. For example, in semi-arid climates, the timing of rainfall is often sporadic [5,6], so the presence of water is not a good indicator of how much water will be available in the future, therefore the optimal response from the plant is unpredictable. In such an environment, bet-hedging strategies are predicted to be advantageous [3,4,7]. Bet-hedging can occur through isogenic plants producing different phenotypes in the same environment, known as diversified bet-hedging [4]. Here, the phenotypic diversity results in a reduced fitness in the optimal environment (because some individuals will have a non-ideal phenotype for this environment). However, over time, the genotype is less likely to go extinct because even when extreme conditions occur, a fraction of the population will survive. By contrast, a genotype that maximizes fitness in the optimal environment would risk extinction under actual conditions when the environment deviates from this.

**Figure 1.**
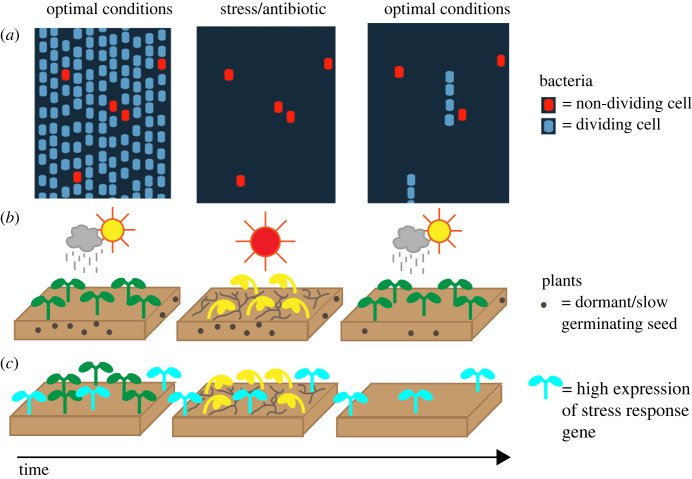
Bet-hedging through phenotypic variability. (*a*) Bet-hedging through phenotypic variability in bacteria. Under optimal conditions, most cells divide normally (blue cells), while a fraction of the population enters a slow growing, non-dividing state that is resistant to antibiotics (red cells). These slow growing cells survive antibiotic exposure, and can switch back into the dividing state upon return to optimal conditions and reconstitute the population. The columns of cells represent columns in a microfluidic device, which is designed to hold bacterial cells in specific vertical channels, allowing them to be tracked over time (inspired by [8]). (*b*) Bet-hedging through seed dormancy or variability in germination times. In optimal conditions, a fraction of seeds germinates. If there is a catastrophic event, such as a drought, the seedlings die but ungerminated seeds can survive. Upon return to optimal conditions, these seeds can germinate. (*c*) Bet-hedging through variability in stress response gene expression. In the absence of stress, abiotic stress response genes can be variably expressed between seedlings [12,13]. This could function as a bet-hedging strategy if plants with higher expression pre-stress are better able to survive the stress.

There are well-studied examples of this in bacteria, where bet-hedging is important for antimicrobial resistance [8–11]. In optimal conditions (i.e. without an antibiotic), a fraction of the population of clonally related cells can enter a slow-growing state that can survive antibiotics. The presence of these cells slows down the average growth of the whole population in optimal conditions. However, if the antibiotic is encountered, these cells will survive, while the rest of the population will be killed (figure 1*a*). These persistent cells can then switch back into a fast-growing state and reconstitute the population [8]. Work on a number of bacterial systems has shown that the phenotypic variability between isogenic individuals is generated through genetic circuits that amplify stochasticity inherent in gene expression [10,14,15]. Work in plants suggests that similar circuits also exist in this multi-cellular context and generate phenotypic variability between individuals [16–19]. How the circuits regulating plant development are influenced by noise and their precise role in generating phenotypic variability is less-well understood than in bacteria. In this review article, we will first use germination as a model system to discuss bet-hedging in plants, before speculating on the functions and mechanisms of variability during later stages of plant development.

## 2. Bet-hedging and variability in germination time

The best-studied example of bet-hedging and phenotypic variability in plants is seed dormancy and germination time. In the 1960s, theoretical work predicted that in unpredictable, randomly varying environments, it is beneficial for long-term population growth rate if a fraction of the seeds from a batch remains dormant in the soil [4,20,21]. The dormant fraction of seeds does not germinate when exposed to permissive conditions for germination, but can go on to germinate at a later date (figure 1*b*). With this strategy, if the environment is unfavourable for plant growth in a given year, for example if a drought occurs during the growing season, there will still be a population of seeds in the soil that can survive this (analogous to antibiotic-resistant bacteria) and then germinate upon return to permissive conditions, preventing extinction.

What is the exact nature of the phenotypic variability in germination time that could operate as a bet-hedging strategy? There are at least two different (but probably often co-occurring) ways that variability in seed germination can manifest. One possibility is that germination times of seeds from the same parent plant vary within a growing season. Another is that germination is spread across years (i.e. between growing seasons) if a fraction of seeds remains dormant in the seed bank and only germinates when favourable conditions for that species' germination return the following year. Both types of variability were carefully characterized using *Lobelia inflata,* a species that grows in disturbed sites (for example in cut forests and along paths) across the east of North America*.* Seeds were harvested from wild or growth-chamber grown plants and then sown in Petri dishes under optimal controlled conditions [22]. Even seeds produced in the first two fruits of the same plant germinated at different times, over a range of 52 days. This species is completely self-fertilizing, and therefore highly inbred and homozygous, thus the variability observed was not owing to genetic variation between seeds in a batch. Seed batches of *L. inflata* that showed high variability in germination times within a season also showed a percentage of seeds that did not germinate under favourable conditions, but did following a cold treatment (which can break dormancy), indicating both types of variability were operating. The strategy of having a fraction of dormant seeds is widespread and has been extensively studied in desert annual species [23–25]. It should be noted that seed dormancy and germination time are plastic (i.e. the fraction of seeds that germinate and the rate of germination is responsive to environmental cues) as well as variable.

**Figure 2.**
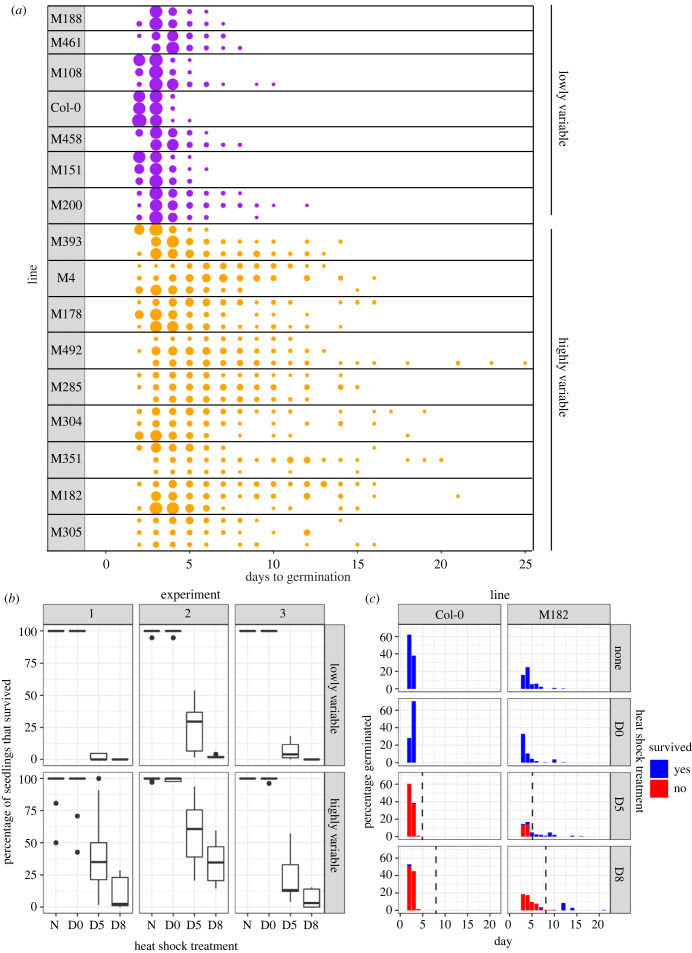
Survival of high and low variability Arabidopsis lines when exposed to a short heat stress. (*a*) Distribution of germination times under controlled conditions for the lowly variable and highly variable lines, first characterized by Abley *et al*. [16] and then used in the bet-hedging experiment. The name of the line is indicated on the left. Each row shows the germination time distribution for one batch of seeds (from a single parent plant) of the given line, the multiple rows for each line show replicate experiments, with independently collected seed batches. Circle size is proportional to the percentage of seeds sown that germinated on a given day. Lines classified (based on their coefficient of variation (CV) of germination time) as lowly variable are shown in purple, those classified as highly variable are shown in orange. Lowly variable lines all have CVs < 0.24, the mean CV of lowly variable lines is 0.17; highly variable lines all have CVs > 0.29, the mean CV of highly variable lines is 0.38. (*b*) Low and high variability lines were exposed to a 30 min heat shock at approximately 49°C either immediately after sowing (D0), or 5 or 8 days after sowing (D5 and D8). The treatment ‘N’ shows non-heat shocked controls. The *y*-axis is the percentage of seeds that germinated which survived until the end of the experiment (25 days after sowing). Experiment 1 included three low variability MAGIC lines, Col-0 (a low variability accession), plus nine highly variable MAGIC lines. Experiments 2 and 3 contained the lines that were present in experiment 1, plus extra lines, so that in total they included seven low variability lines (six low variability MAGIC lines, plus Col-0) and nine high variability MAGIC lines. Lines were partitioned into high and low variability lines according to their average CV across the experiments. (*c*) Germination time distributions and survival, for example low variability line (Col-0) and a high variability MAGIC line (M182). Each row of panels is a different treatment. Bars show the percentage of seeds sown that germinated on a particular day. Colours of bars show the fractions of plants that germinated on a particular day that had survived or died by the end of the experiment (25 days after sowing). The vertical dashed black lines show the timings of heat shocks. The raw data for this experiment and the analysis script used to generate the figures are available as the electronic supplementary material, files.

To illustrate how variability in germination time within a season could function as a bet-hedging strategy, we used a number of genetically distinct lines in the model plant *Arabidopsis thaliana*. These multi-parent advanced generation inter-cross (MAGIC) lines were generated from 19 natural accessions of Arabidopsis [26] and were previously characterized to have high or low variability in germination time, based on the coefficients of variation (standard deviation/mean) of their germination time distributions [16], figure 2*a*. Previous quantitative trait locus (QTL) mapping for variability in germination time within the MAGIC lines revealed genetic regions associated with known loci underlying seed dormancy (including *DOG1*, which will be discussed in a later section), suggesting that variability in germination time within a season and seed dormancy level have a shared mechanistic basis in Arabidopsis [16].

Using MAGIC lines with high and low variability in germination time, we sought to test whether high variability in Arabidopsis seed germination time could be beneficial for survival in suddenly varying environments. The MAGIC lines are highly inbred, and therefore the seeds in a batch are assumed to be almost genetically identical. We collected fresh seed batches from individual mother plants grown in the same environment, stored them for approximately 30 days, and then sowed approximately 150–200 sterilized seeds of each line on agar plates under controlled conditions. We did not use stratification (a cold dark treatment of imbibed seeds prior to sowing which promotes uniform germination). Under these conditions, there was a relatively low level of seed dormancy within the MAGIC lines, allowing us to assess germination time distributions among large populations of germinating seeds. Detailed methods are as described in [16].

To simulate an unpredictable lethal environmental stress, we applied a short heat shock (49°C for 30 min) to high and low variability genotypes, either immediately following sowing in non-stressful growing conditions, or 5 or 8 days after sowing. This was done by sealing the agar plates containing seeds and seedlings with parafilm and floating them on a heated water bath. This treatment kills seedlings but does not damage ungerminated seeds (figure 2*b*, ‘N’ treatment (no heat shock) compared with heat shock on day 0) [27]. We scored germination each day and followed the seedlings that germinated until 25 days after sowing, to see if they died (i.e. turned white) or survived (remained green) following the stress treatment. We found that in higher variability lines, a greater proportion of seedlings were alive at the end of the experiment when a heat shock was applied at day 5 or day 8 (figure 2*b*). This is because with low variability lines, most seeds germinated before day 5, and these seedlings then tended to be killed by the heat shock (e.g. figure 2*c,* Col-0). In higher variability lines, the majority of seedlings that germinated before the heat shock were killed but late germinating seeds were still available to germinate after the stress (figure 2*c*, M182). These findings illustrate that having higher variability in germination time can be advantageous by allowing a fraction of the population, which would have germinated later than the heat-stress occurred even in its absence, to survive a stress treatment as seeds and then go on to germinate at a later time.

This is a demonstration of how bet-hedging through variability in germination time could lead to increased survival in a population of individuals. However, to provide evidence that germination time variability functions as an adaptive bet-hedging strategy in natural populations, it is necessary to show that naturally occurring variation in germination time variability has a genetic basis, that the level of variability is correlated with unpredictability of the natural environment and that it confers a fitness benefit under naturally occurring conditions [4]. As mentioned above, work in Arabidopsis has shown that variability in germination time and the level of seed dormancy is under genetic control [16,28–31]. Some of the strongest evidence for variability in germination time functioning as a bet-hedging strategy comes from long-term observations of a number of species in the Sonoran desert. It has been shown that among these species, higher levels of seed dormancy correlate with higher between-year variation in reproductive success, which itself correlates with greater sensitivity of reproductive success to variation in precipitation [25]. The degree of within-season and between-season variability in germination times fits with predictions for the optimal germination strategy in this variable environment [23,32].

In contrast to wild plant species, in agriculture, variability in germination time desynchronizes the establishment and growth of crop plants, and therefore tends to be undesirable (reviewed in [33]). Crop species have been artificially selected for rapid germination and low dormancy levels and pre-treatments of seeds, called priming, are used to promote germination uniformity [33]. Farmers need a predictable rate of germination to achieve an optimal seedling density, which directly relates to final yield. For vegetable crops, it is also important that seedlings emerge at the same time so at the point of harvest all the crops are of big enough size to be high value. For example, variability in the timing of seedling emergence in lettuce is problematic as later germinating seeds give rise to plants that mature later and are much smaller at the time of harvest (owing to exponential growth which means that a few days difference in the period of growth will have a large impact on final plant size) [34], thus reducing yield and profits and making crop management difficult [35]. Understanding the mechanisms underlying bet-hedging strategies could be important for developing crops with more predictable germination times even when faced with increasingly unpredictable environmental conditions owing to climate change [7,36]. We next turn towards the mechanisms underlying variability in germination time, with a focus on Arabidopsis, where much of our understanding has been developed.

## 3. Mechanisms regulating variability in seed germination time

Variability in Arabidopsis germination time can occur between developmentally and otherwise phenotypically equivalent seeds. In lines with high variability in germination times, the full range of germination times was found to be generated by seeds within a single seed pod, and the germination time was independent of the position of the seed in the pod [16]. This suggests that mechanisms must exist to generate differences between seeds that develop side-by-side in the same fruit. In Arabidopsis, variability in seed germination time (i.e. the spread of germination times among seeds that do germinate) and seed dormancy level (the ability of seeds not to germinate under optimal conditions) are likely to be controlled by overlapping mechanisms [16,37]. Arabidopsis exhibits physiological seed dormancy, the most common form of seed dormancy, which uses hormones to inhibit germination [38]. Below, we first briefly outline some of the key molecular processes involved in controlling physiological seed dormancy and germination, and then go on to discuss models for how they could generate differences between genetically identical seeds developing in the same seed pod. For a more detailed and comprehensive explanation of the potential mechanisms of variability in germination time, please see Sharma & Majee, 2023 [37].

Processes operating throughout the lifetime of a seed, influenced both by the maternal environment and that which the seed finds itself in, have an impact on whether and under what conditions the seed will germinate. The plant hormone abscisic acid (ABA) is important for regulating dormancy and germination during all stages of seed development [39]. Seeds developing on the mother plant and newly shed dry seeds have high levels of ABA, the levels of which depend on the maternal environment. Once seeds are shed from the parent plant, if they are stored in dry conditions, their level of dormancy drops in a process called dry after ripening. During this phase, ABA levels appear to remain high [40]. When seeds are then imbibed in water, ABA levels drop, but dormant seeds sustain higher ABA levels than those which go on to germinate [39,41].

A large network of regulatory factors centres on ABA and another hormone, giberrellic acid (GA), which promotes germination. Increases in GA biosynthesis, which are triggered upon imbibition, are important for germination to occur [40,42,43]. The signalling pathways of ABA and GA converge to oppositely regulate the activity of a set of transcriptional regulators which inhibit germination. ABA upregulates ABA-INSENSITIVE 4 and 5 (ABI4, ABI5) and DELLA proteins, thus inhibiting germination, while GA downregulates these factors, promoting germination [18,19,44–46]. Via their influence upon these transcriptional regulators, ABA and GA influence the expression of their own and each other's biosynthetic and catabolic enzymes [18,19,47,48]. The sum of these interactions creates a mutual antagonism between the two hormones, where high levels of ABA inhibit the accumulation of GA and vice versa [49,50]. Environmental signals feed into this network and alter the ABA/GA balance to influence the decision to germinate (reviewed in [49]).

Other genes with important roles in regulating germination and seed dormancy have been identified through QTL mapping in Arabidopsis, including those underlying the *DELAY OF GERMINATION* (*DOG*) loci [28]. *DOG1* is an important determinant of the degree of primary seed dormancy (that which is established when still developing on the mother plant) in Arabidopsis and is expressed during seed development on the mother plant [51] and following imbibition [51]. *DOG1* expression is influenced by maternal temperature [52], however how this regulation occurs is poorly understood [39]. Mapping and characterization of the *DOG1* gene revealed that it feeds into the ABA signalling pathway to increase ABA-sensitivity and inhibit germination, independently from the presence of ABA [53–55]. It does this through inhibiting ABA-HYPERSENSITIVE GERMINATION 1 (AHG1) and AHG3, two clade A PP2C phosphatases, which negatively regulate ABA signalling and dormancy [54,55]. This both activates the ABA signalling pathway downstream of ABA and, by removing the block on ABA signalling caused by PP2C phosphatase activity, increases the sensitivity to ABA. The levels of DOG1 protein in freshly harvested seed are correlated with the levels of dormancy [56] and the expression of *DOG1* is regulated at multiple levels [49]. Whether *DOG1* primarily influences primary dormancy levels prior to sowing, or the decision to germinate after sowing, or both, is unknown [39].

Recently, a new mechanism of germination-regulation was discovered that may function independently of ABA/GA to regulate germination following imbibition, particularly under conditions where water is limiting. A prion-like protein, called FLOE1, was found to be highly expressed in dry seeds and undergo a reversible phase separation as seeds transition from the dry to the imbibed state containing water [57]. In dry seeds, or those that have been imbibed but subsequently experience osmotic stress, the protein is distributed diffusely throughout the cytoplasm and inhibits germination. In the presence of water, it forms condensates, which are visible as large particles in the cytoplasm when imaging *FLOE1::FLOE1:GFP*. The formation of condensates seems to prevent FLOE1 inhibiting germination, thus allowing germination to proceed when water is available. Thus, it appears that the water-dependent phase-changes in FLOE1 are a way for germination to be regulated according to water availability, preventing germination under drought conditions, but allowing it to occur again if water becomes available. The authors investigated whether natural variation in *FLOE1* expression could function as a bet-hedging strategy. Perhaps unexpectedly based on the role *FLOE1* seems to play in drought stress, its expression across natural accessions is correlated with lower temperatures and wetter summers in the accessions' habitats. Expression of a splice isoform, *FLOE 1.2*, which is associated with larger condensate formation and increased percentage germination, showed a negative correlation with between-year variability in rainfall. This could indicate that lower expression of this version (and thus reduced percentage germination) has been selected as a bet-hedging strategy in unpredictable conditions [57].

The work summarized above has revealed important mechanisms involved in controlling the level of seed dormancy and the decision to germinate. But how do ABA, GA and other regulatory factors generate differences between isogenic seeds produced from a single plant? When seeds are shed from a parent plant and exposed to permissive conditions for germination, why do all the seeds not germinate at the same time, if they all carry the same genetic networks controlling germination and dormancy?

One possibility is that stochastic processes operating within the networks that regulate germination and dormancy could be involved in generating differences between seeds, as has been proposed for generating phenotypic diversity in microorganisms [10,11]. Stochasticity could in principle play a role at virtually any stage of seed development and germination. It is possible that the processes of seed dormancy establishment under the influence of maternal cues, or the process of dormancy reduction during dry after-ripening varies from seed to seed in a stochastic manner. Other sources of variability between seeds could be in the speed of making the decision to germinate following imbibition or in the subsequent growth of germinating seeds [33]. Once the level of dormancy has dropped, for example through dry after-ripening, all seeds in a batch may be able to germinate, but there may still be variation between them in the speed of germination. The early stages of germination following water uptake involve the translation of proteins from transcripts that have been stored in dry seeds [58]. This is followed by sequential waves of new transcription [7]. Both transcription and translation are subject to stochastic effects owing to the low copy numbers of molecules involved [59], which might influence germination speeds. Although all of these processes could be influenced by stochasticity and generate variability between seeds, the extent to which this is the case and correlates with variability in germination time is unclear. However, a number of mathematical models of germination time variability which incorporate stochasticity in some form have been developed. How stochasticity plays a role in generating variable germination times varies between the models.

One type of model, called the population-based threshold model, assumes that within a population of seeds, there is a distribution of sensitivities to hormones and environmental factors and these different sensitivities underlie the variability in germination time [60,61]. Although not explicitly modelled, the distribution of sensitivities in the population has been hypothesized to be established through stochastic effects in gene expression that are amplified through positive feedback to give rise to differences between individuals [62]. The population-based threshold model has been successful in explaining a wide range of observations about the timing of germination and its response to treatment with regulatory factors. In this model, each seed's sensitivity threshold equates to the basal level of a regulatory factor that is needed to promote germination. The time at which a seed will germinate is assumed to be inversely proportional to the difference between the level of the regulatory factor and the sensitivity threshold. Seeds that have a low sensitivity threshold (i.e. are highly sensitive), germinate sooner because the difference between the level of the factor that is promoting germination and that seed's sensitivity threshold is larger. It is assumed that seeds germinate when this difference multiplied by time reaches a constant, i.e. seeds germinate when they have accumulated enough regulator-time. Thus, if the level of the germination regulator is increased, more seeds will be able to germinate (more of their sensitivity thresholds will be exceeded) and the time window within which the germination of these seeds is completed will be smaller. The model can account for the observation that a batch of seeds which appears to be highly variable when there is a low concentration of GA applied, may germinate within a small time-window when a high concentration of GA is applied, and this arises from the model even assuming the same underlying distribution of sensitivities [60].

Another type of model explicitly models the feedbacks involved in amplifying stochasticity to give rise to differences between seeds [16,17,63]. These models hypothesize that stochasticity in the networks controlling the levels of regulatory hormones generates variability in germination timing. One model can capture variability in ABA levels and therefore in germination propensity, through feedback regulation on ABA [17]. It is assumed that ABA regulates its own levels via the production of two intermediate molecular species, one of which promotes ABA biosynthesis and one of which promotes ABA catabolism. This is backed up by microarray data which provides evidence that ABA promotes the expression of several genes involved in its biosynthesis, as well as the ABA catabolic enzyme CYP707A2 [63]. In stochastic simulations of these interactions, it is shown that by varying different parameters, either individually or in combination, the level of noise in ABA levels can be tuned in different ways with respect to the mean ABA level, including the possibility that variability in ABA levels varies independently of the mean ABA level [17]. Therefore, simulations show that, in principle, the network controlling ABA-regulation could be selected, or manipulated, to be more or less variable without necessarily having an effect on mean ABA levels.

This stochastic ABA feedback model has been tested by using data from an Arabidopsis line where ABA biosynthetic enzymes are driven under the control of an ABA-dependent promoter [64]. This increases the positive feedback of ABA on its own synthesis, which in the model is predicted to increase ABA levels and decrease the noise in ABA levels [17]. This decrease in noise of the ABA levels is because as the positive feedback increases, the levels of the intermediate factor which promotes ABA biosynthesis increase and at higher molecule numbers this intermediate is less affected by stochastic fluctuations that impact on the variability of ABA levels. The experimental data show that lines with the increased positive feedback have higher levels of ABA and reduced between-seed variability in ABA levels, consistent with the model's prediction [64]. Also, the data show that seeds with the positive feedback on ABA biosynthesis have lower variation in germination proportion between batches of seeds.

Another type of stochastic model considers the mutually antagonistic interactions between ABA and GA, which form a bistable switch [16,63]. This model captures the transition of seeds from a high ABA/low GA state when they are sown, to a state with low ABA/high GA, which allows them to germinate. When this network is modelled stochastically [16], it captures differences in variability of germination time seen between different Arabidopsis genotypes. It can account for low variability in germination time by assuming that in lowly variable lines the parameters of the system are such that it operates in a monostable regime, where, following sowing, only the low ABA/high GA state is stable (for example, if the parameter for ABA sensitivity is set to a low value) and all seeds transition to this state relatively quickly. To account for higher variability in germination time, the model assumes that highly variable lines operate within the bimodal regime of the model. In this case, following sowing, both the high ABA/low GA (non-germinating) state and the low ABA/high GA (germinating) state are possible. Representing experimental data, the simulations are initialized with seeds in the high ABA/low GA state. The transition between the non-germinating and germinating states is influenced by stochastic fluctuations, therefore the time at which individual seeds transition is variable, giving rise to highly variable germination times. An increase in sensitivity to ABA causes the system to be in the bistable regime and can increase the stability of the high ABA/low GA steady state. Both effects result in an increase in the level of variability. This was related to data from QTL mapping which detected two QTL peaks underlying variability, both of which contained genes with roles in ABA sensitivity (*ANAC060* for one QTL and *AHG1* and *DOG1* for the other [54,65–67]), which when mutated had an effect on the level of variability in germination time [16].

The stochastic models of [16] and [17] treat the seed as one compartment and do not consider the impact of cellular-level processes on decision-making. If the noisy circuits involved were modelled at the level of multiple interacting cells, it is possible that stochastic effects at the level of individual cells would be averaged out over the multiple cells constituting a seed. However, ABA and GA signalling, synthesis and degradation occur in small, distinct, subsets of cells in the embryonic root, where the germination process is initiated, as well as in the surrounding endosperm [37,63]. Given the relatively small number of cells in which the decision is made, it is possible that stochastic effects are not averaged out. The spatial distribution of ABA and GA signalling and metabolism was shown to have a significant effect on decision-making in deterministic simulations of the ABA-GA network in response to varying temperature inputs, compared with a model without the spatial distribution [63]. Thus, it would be interesting to generate a cellular-based stochastic model of the ABA-GA network and test the impact of the experimentally observed spatial distribution of ABA and GA signalling components.

An alternative source of variability in germination time could come from micro-environmental differences. It is possible that some genotypes of plants produce seeds that are more sensitive to these differences and thus amplify noise in the external environment [68,69], as well as, or instead of, noise in their internal environment. In a study of variability in seed germination time among isogenic seeds of *L. inflata*, it was found that the positioning of seeds within Petri dishes and the positioning of the Petri dishes within a growth chamber with apparently uniform conditions accounted for around 30% of the variance in germination time [22]. This suggests that sensitivity to micro-environmental gradients within the growth chamber and Petri dishes contributed significantly to the variability observed.

## 4. Testing models of germination time variability

To further investigate the mechanisms underlying germination time variability and to test the different models that have been proposed, it is important to be able to quantify differences between seeds at the molecular level and ideally relate these molecular-level differences between individual seeds to their germination times. ABA levels can be quantified in individual seeds using liquid chromatography-electrospray ionization-tandem mass spectrometry [70]. Measurements of ABA levels in individual seeds from the same silique showed almost threefold variation between individual seeds. It would be interesting to compare the levels of between-seed variability in ABA levels for Arabidopsis lines with high and low variability in germination time (for example those shown in figure 2*a*).

An important step forward in quantifying variability at the molecular level in seeds was made recently, with the publication of a high throughput method for performing RNAseq on individual seeds [71]. The authors obtained transcriptomes for hundreds of seeds, across a range of conditions, including seeds 1 h after sowing in permissive conditions for germination and at multiple time points under conditions that induce secondary dormancy (SD). SD involves a reinstatement of the dormancy programme following seed sowing in unfavourable conditions. Samples were collected for 1, 3, 5 and 7 day SD treatments, and 1 day after release from a 7 day SD treatment into permissive conditions. The authors assessed the technical variability of their method by using a pool-split control where single-seed extracts were mixed (thus homogenizing them) and then split into separate samples and processed along with other samples. The authors identified highly variable genes in relation to the pool-split control. The number of highly variable genes decreased with increasing time under the secondary-dormancy inducing conditions and the highest number of highly variable genes was found 24 h after release from the 7 day SD induction treatment, as seeds were nearing the time that they would germinate. At this time point, translation-associated genes were variably expressed between seeds. Interestingly, highly variable genes were also found as early as 1 h after sowing in permissive conditions, suggesting that variability is present at early time points before the decision to germinate has been made. In all of the time points, the variability in transcriptomes between seeds was higher than in the pool-split control, showing that the technique is able to detect biologically relevant variability.

How can such transcriptomic data be used to elucidate the mechanisms underlying variability in germination time? The genes that are highly variable 24 h after release into permissive conditions may not represent those whose variability between seeds is causative for the decision to germinate, but may rather be downstream of the decision to germinate. In order to gain a mechanistic understanding, it may be informative to further investigate genes that are highly variable early on following sowing in permissive conditions (for example those in the 1 h after sowing time point, or at shorter time intervals after transfer from SD inducing conditions to permissive conditions). It would then be possible to use genetic perturbations to test whether these highly variable genes play a role in generating variability. Additionally, Krzyszton *et al*. [71] used the Col-0 accession, which tends to have relatively rapid and uniform germination, but induced greater differences between seeds with the SD treatment. Going forward, it could be informative to perform single-seed RNAseq on lines with high and low variability in germination time [16] and identify transcripts that are highly variable at different time points following sowing in permissive conditions. Additionally, it would be interesting to know when variability in regulators of germination is first present. Early on following water uptake to the dry seed, stored gene transcripts are translated. Prior to sowing, during the after-ripening process, the levels of these stored transcripts of dormancy promoting genes are downregulated over time, leading to a reduction in seed dormancy [72]. Thus, one possibility is that variability in these stored transcripts between dry seeds could create variability in germination time.

One limitation of both the mass-spectrometry used for the ABA measurements and the single-seed RNA seq method is that they are inherently destructive, so it is not possible to relate the gene expression profile or hormone levels of a seed with its germination time, or to follow a seed from the early time points after sowing to later time points (also discussed in [7]). An ideal way to test potential regulators of germination-time variability would be through single-seed time-lapse imaging. Fluorescence or bioluminescent reporters for candidate genes thought to be involved in decision-making could be followed throughout time and correlated with the seed's decision to germinate or not and with its germination time. Fluorescence resonance energy transfer-based biosensors for ABA and GA offer the potential to image GA and ABA dynamics over time at the single-cell level in individual seeds [73–75], which could help to reveal the cellular basis for between-seed variability. However, the opacity of the seed coat currently obstructs our ability to perform live imaging of intact seeds [7].

## 5. Bet-hedging in plant development: going beyond germination

In later stages of plant development, there is a growing body of work revealing phenotypic variability between isogenic plants that could function as a bet-hedging strategy [68]. Because these later stages are more accessible than seeds for live time-lapse confocal imaging, they represent opportunities for in-depth studies of the molecular mechanisms underlying between-plant phenotypic variability.

QTL mapping studies have shown that post-germination plant traits exhibit phenotypic variability, the level of which is under genetic control. The level of between-plant variability in a number of adult plant traits (rosette leaf number, rosette diameter, plant height, the number of branches) was quantified under controlled environmental conditions for a large number of highly inbred *Arabidopsis* lines [76]. QTL mapping revealed a number of loci associated with the level of variability in each trait, many of which overlapped with those controlling the mean value of the trait, however three QTL were specific for variability. This suggests that the level of variability could be selected for, independently from the mean trait value, indicating that there may be regulatory mechanisms that specifically regulate variability. Variability in glucosinolate defence compounds between plants was also shown to have a genetic basis, independently from the mean glucosinolate level [77]. These compounds protect plants from herbivory and pathogens, and tend to promote counter-adaptation of resistance to the compounds in the herbivore and pathogen species. Thus, variability between plants could be advantageous to reduce the selective pressure for this counter-adaptation [77].

Genetically identical plants growing in the same environment also show variability in expression levels of some genes, which could underlie their phenotypic variability. Using microarray data, gene expression variability was quantified between independent pools of plants for different genetic lines of a recombinant inbred population [77]. A number of expression QTL (eQTL) were identified that affect gene expression variability of other loci, independently from transcript mean abundance. *ELF3*, a circadian clock gene, was found to probably underlie one of the eQTL associated with global gene expression variability. Different natural alleles of *ELF3* were shown to influence variability in expression of circadian clock genes as well as variability in glucosinolate accumulation and flowering time [77].

An important step forward in understanding the extent of between-plant variability and for which aspects of plant function variability might be particularly important, came with the characterization of transcriptional variability at the single seedling level in Arabidopsis [12,78]. In contrast to traditional RNA sequencing methods of obtaining RNA from multiple plants pooled together, Cortijo *et al*. [12] collected 14 single Arabidopsis seedlings every 2 h of the day (12 time points) and performed RNA sequencing on the individual plants. The transcriptomes obtained revealed differences in gene expression between the isogenic seedlings growing in the same environment, which were not apparent in previous studies that averaged across multiple plants. Nine per cent of expressed genes were categorized as being highly variable with respect to the rest of the transcriptome. Highly variable genes were enriched for gene ontologies associated with the response to biotic and abiotic stresses, with specific gene ontologies being enriched during the day or night. By contrast, lowly variable genes were enriched for gene ontologies associated with primary metabolism. Highly variable genes tended to be smaller than lowly variable genes and their promoters had a higher number of transcription factor binding sites. They were also enriched for histone modifications associated with a compacted chromatin environment [12]. Together these results indicate specific mechanisms of regulation are associated with high transcriptional variability, supporting the idea that mechanisms may have evolved to specifically regulate the level of variability in gene expression.

**Figure 3.**
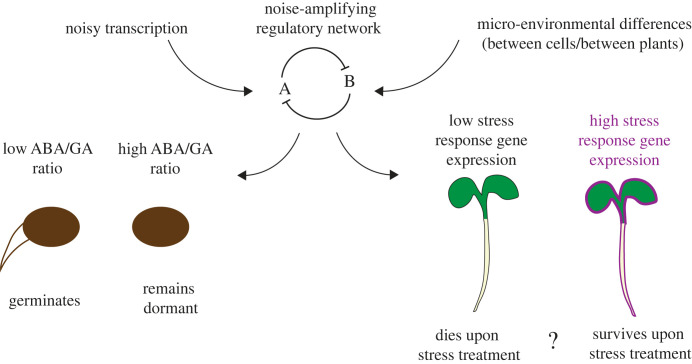
From noisy gene expression to between-individual variability. Particular gene regulatory network architectures can amplify noise, either coming from the stochastic nature of transcription initiation, or from microenvironmental differences. Left: in the case of seed germination, the hormones ABA and GA mutually antagonise each other, which creates a bistable switch. Noise in the network has been proposed to generate between-seed differences in the dynamics of the ABA/GA ratio, underlying differences in germination time and whether a seed germinates or not [16]. Right: stress response genes have been found to be variably transcribed between cells in the same tissue owing to stochastic transcription initiation [80,81]. Some stress response genes are also variably expressed between individual seedlings [12,13]. This could function as a bet-hedging strategy by allowing a fraction of plants to have high stress response gene expression even in the absence of stress, which could promote survival following exposure to stress. How noisy transcription which generates cellular-level variability is related to the generation of variability at the between-plant level is unknown.

Single plant transcriptomics has also been performed for inbred maize plants in a field environment [13]. Genes identified as being highly variable between plants were largely involved in similar pathways to those identified in laboratory conditions in Arabidopsis [12]. For example, genes related to response to biotic and abiotic stresses, photosynthesis, cell wall organization, secondary metabolism, brassinosteroid metabolism, and response to hormones such as cytokinin, ABA, jasmonic acid and GA were highly variable between plants [13]. Interestingly around 14% of the transcriptome showed a spatial pattern of expression levels across the field and there were also spatial patterns in metabolome and phenotype data. This suggests that responses of plants to micro-environmental differences in the field were responsible for some of the between-plant variability observed. One question is whether the genetic networks involved in regulating the expression of highly variable genes are particularly wired to sense and amplify micro-environmental differences to give rise to different states of gene expression. This could be the case if the networks contain architectures known to be capable of amplifying noise to generate diverse output states, for example positive feedback loops [79] (figure 3).

The studies described above have identified a number of genes, phenotypes and pathways that are highly variable between plants, but, unlike germination, evidence for a functional role of the variability in later stages of plant development is currently lacking. One hypothesis is that variable expression of stress responsive genes [12,13] could function as a bet-hedging strategy [68] (figure 1*c*). According to this hypothesis, differences between plants in how they activate stress response pathways may prepare a subset of plants to respond to stress while they are still in the unstressed state. In the event of stress, the plants with the stress response pathways already switched on may be more likely to survive the stress and regrow once optimal conditions return (figure 1*c*). In support of this hypothesis, following stress treatments such as heat-shock or salt stress, for some stress levels, a fraction of isogenic plants survive, suggesting between-plant differences in survival ability [27,82].

To test whether highly variable genes function as part of a bet-hedging strategy, live imaging could be used to test whether their expression prior to stress predicts survival following stress exposure at the individual seedling level. To qualify as a bet-hedging strategy, it must be shown that there is also a cost to high expression of the stress responsive genes in the non-stress environment. One possibility is that there is a trade-off between growth and stress response in plants, similar to findings in bacteria [83]. For example, in plants, DELLA proteins act as a hub to integrate between several hormonal pathways controlling plant development and stress response [84]. Their activity confers stress response, but inhibits growth [82], suggesting such a trade-off may apply to plants and explain why a bet-hedging strategy of variable gene expression and growth rate may be advantageous over having uniformly high activation of stress response pathways across all seedlings [82].

If, as discussed above, transcriptional and phenotypic variability between plants is functionally important, how is this variability generated at the cellular and molecular levels? One hypothesis is that, similar to the case proposed for seeds, regulatory networks controlling highly variable genes act to amplify the inherently stochastic processes involved in gene expression, and/or microenvironmental differences between plants (figure 3). Advances in the field of gene expression quantification using live imaging provide tools to measure gene expression in seedlings in a way that can inform us about these processes. Araújo *et al*. [85] used a dual reporter system to measure the levels of intrinsic and extrinsic noise, as was first performed in *Escherichia coli* [86]. This work showed that, similar to in single-celled organisms, gene expression in plants is noisy, owing both to stochastic transcription initiation (intrinsic noise) and between-cell variability in gene expression states (extrinsic noise), even for the relatively highly expressed constitutive promoters that the authors focused on.

Two recent studies extended measurements of noise in gene expression to environmentally responsive genes involved in heat-stress and phosphate response [80,81]. Both used a nascent messenger RNA labelling technique to measure the rates of transcription initiation at specific genomic loci in individual cells. The technique makes it possible to capture the real-time activity of RNA polymerase II at specific promoters [80]. During heat stress application, there was intercellular variability in transcriptional activation of the heat stress responsive genes, *HEAT SHOCK PROTEIN 101* (*HSP101)* and *HEAT SHOCK TRANSCRIPTION FACTOR A2 (HSFA2)*. Previous bulk studies that averaged over the whole tissue showed that the average level of these genes' expression increased following heat stress. Characterizing this at the single-cell level revealed that, for each gene, heat shock caused a subset of cells to switch to increase its transcription, while a fraction of cells remained transcriptionally off [80]. The average rate of transcription remained approximately constant for actively transcribing cells, and thus the tissue-wide response of an increase in average expression was owing to a change in the fraction of actively transcribing cells. Hani *et al*. [81] studied intercellular variability in RNA transcription initiation for the phosphate response gene *SPX1*, which is expressed in low-phosphate conditions and inhibited upon phosphate resupply. They also detected a high level of between-cell variability in the downregulation of *SPX1* promoter activity in response to phosphate addition, with neighbouring cells in the same root showing very different rates of transcription initiation. Thus, between-cell variability in plant gene expression was clearly revealed for the first time for environmentally sensitive genes.

However, despite this evidence that gene expression can be variable at the cellular level in plants, it remains unclear how between-cell variability is related to between-plant differences in transcription such as those that have been found for some stress response genes [12,13]. We might assume that stochastic activation or inactivation of a gene's expression occurs with a particular probability that will depend on the regulatory network and environmental inputs influencing that gene. If this stochastic activation occurs over many individual cells, it could tend to give a consistent fraction of active versus inactive cells in every plant. This would result in low levels of variability in gene expression between whole plants. This fractional cellular response has been modelled for the flowering inhibitor, FLC, where an epigenetic polycomb-based bistable switch has been proposed to control *FLC* expression state at each individual locus [87]. In this system, at the whole plant level, *FLC* expression is inhibited by prolonged cold in such a way that expression levels are a quantitative read out of the duration of winter cold experienced by the plant. At the cellular level, the flipping of this epigenetic switch at individual *FLC* loci is proposed to be stochastic and probabilistic, with the probability of *FLC* being switched off being influenced by the length of winter cold. Thus, according to this mechanism, although the behaviour at the cellular level is stochastic, all plants that have experienced the same environmental conditions would end up with a very similar fraction of *FLC* ‘on’ and ‘off’ cells, which allows an accurate response to the environment at the cell-population level, accurately coupling flowering time to the environment. In this case, *FLC* inhibits the expression of a downstream mobile protein, called FT, which moves through the phloem to the shoot apical meristem and promotes its transition to flowering [88]. Because of its mobility, FT can probably function as a read out of the average *FLC* activity across the plant [89]. Thus, heterogeneity at the cellular level in *FLC* activity could lead to accurate whole-plant levels of FT.

Given that cellular-level heterogeneity arising from noisy transcription does not necessarily lead to whole-plant level heterogeneity, how can variability between plants be generated? One possibility, as mentioned previously, is that noise-amplifying feedback loops regulating gene expression also amplify micro-environmental differences between plants. For example, for environmentally responsive genes that are highly variable between plants [12,13] this could create between-plant differences in the number of cells that activate transcription, even under apparently uniform environmental conditions. Another possibility is that positive feedbacks involving mobile cues, such as hormones and metabolites, amplify stochasticity and give rise to between-plant heterogeneity. Such a mechanism might underlie the observed between-plant variability in glucosinolate defence compounds [77], metabolites which are themselves highly connected within transcription factor and hormone-regulatory networks [90]. For example, for a given gene or metabolic pathway, once a fraction of cells is stochastically activated, this could trigger positive feedback through a mobile signal which would serve to activate transcription of the gene or pathway in cells throughout the tissue or whole plant. This could result in variability between plants in whether the tissue or plant as a whole switches ‘on’ or remains ‘off’ and variability between plants in the timing at which they switch ‘on’. Using a combination of quantitative live imaging techniques that link the cellular, tissue and whole-plant levels will allow us to test these possibilities.

In the future, it will be important to test hypotheses about the roles of specific genetic circuits in regulating the variability in expression of highly variable genes. To do this, quantification of gene expression variability at the cellular, tissue and whole-plant levels could be carried out in both wild-type and mutant backgrounds for the pathways of interest. This could reveal which regulatory elements are important for controlling cellular-level dynamics and variability in gene expression, and what the consequences are for gene expression and the phenotype at the whole plant level if variability at the cellular level is altered.

## 6. Conclusion

Germination is an excellent model system for testing theoretical predictions about bet-hedging evolving in unpredictable environments. It is the plant phenotype with the most obvious phenotypic variability and a clear functional relevance. A number of mechanistic models have attempted to explain the generation of variability in germination time. Testing the models is currently somewhat difficult because quantifying variability in seeds at the molecular level through live imaging is not currently possible. However, recent advances in single-seed RNA sequencing raise new possibilities for comparing model predictions with experimental data. In later stages of plant development, advances in gene expression quantification using live imaging make it possible to measure noise in gene expression in a way that is not possible in seeds. There is evidence for variability in plant phenotypes beyond germination and for gene expression variability between plants, including for stress responsive pathways, which might function as a bet-hedging strategy. Future work is needed to test whether variability in seedling and adult plant traits acts as a bet-hedging strategy. Additionally, how variability in gene expression between cells links to variability in gene expression and phenotypes between plants remains poorly understood.

## Data Availability

The raw experimental data and the analysis script used to generate the figures are available as electronic supplementary material files [91].
